# *β*-Sitosterol Alleviates Inflammatory Response via Inhibiting the Activation of ERK/p38 and NF-*κ*B Pathways in LPS-Exposed BV2 Cells

**DOI:** 10.1155/2020/7532306

**Published:** 2020-05-27

**Authors:** Yanan Sun, Ling Gao, Wei Hou, Jie Wu

**Affiliations:** ^1^Department of Radiation Oncology and Therapy, The First Hospital of Jilin University, Changchun 130021, China; ^2^Jilin Provincial Key Laboratory of Radiation Oncology and Therapy, The First Hospital of Jilin University, Changchun 130021, China; ^3^NHC Key Laboratory of Radiobiology, School of Public Health, Jilin University, Changchun 130021, China

## Abstract

Neurodegenerative disease is a disease state in which neurons in the spinal cord and brain are lost. Studies show that sustained neuroinflammatory reactions release toxic factors, damage neurons, and lead to neurodegenerative diseases. Therefore, inhibiting neuroinflammation may be an effective measure to alleviate neurodegenerative diseases. Microglia is an important participant in the neuroinflammatory response. *β*-Sitosterol (BS) is widely found in various vegetable oils, nuts, and other plant seeds. Studies have found that BS has a wide range of anti-inflammatory effects in peritoneal macrophages and other peripheral tissues. However, no studies have reported the effect of BS that impacts microglia activity. Herein, we further study the effect of BS on impacts microglia activity. Firstly, BV2, a murine microglial cell, was treated with different concentrations of BS prior to stimulation of LPS, and then the inflammatory mediators and the expression of related signaling molecules were tested. The inflammatory response results illustrated that BS treatment can reduce the LPS-induced expression of inflammatory mediators (interleukin-6 (IL-6), inducible nitric oxide (iNOS), tumor necrosis factor-*α* (TNF-*α*), and cyclooxygenase-2(COX-2)). The related signaling pathway analysis demonstrated that BS treatment can inhibit the LPS-induced activation of p38, ERK, and NF-*κ*B pathways. To sum up, the results demonstrated that BS impacts microglia activity via repressing the activation of p38, ERK, and NF-*κ*B pathways.

## 1. Introduction

The spinal cord and brain, composed of neurons, have different functions such as processing sensory information, controlling movement, and making decisions. The loss of neurons can lead to neurodegenerative diseases such as Alzheimer's (AD), Huntington's disease (HD), and Parkinson's disease (PD), which seriously affect the quality of human life [1, 2]. However, currently, there is no effective way to treat such diseases. Numerous studies have confirmed the truth that inflammation plays a vital role in the onset process of diseases [3, 43. When neuroinflammation occurs, immune cells are activated to release toxic factors such as inducible interleukin-6 (IL-6), cyclooxygenase-2 (COX-2), tumor necrosis factor-*α* (TNF-*α*), and nitric oxide (iNOS) and damage to peripheral neurons, which in turn can further activate immune cells, and so repeatedly, a vicious cycle is formed, eventually leading to such diseases [5–7]. Therefore, inhibiting neuroinflammation may be an effective measure to alleviate neurodegenerative diseases.

Microglia is resident immune cells in the brain and is a type of glial cells. When the brain is damaged, microglia responds quickly and removes harmful substances from the central nervous system [8, 9]. However, excessively activated microglia or persistent microglia inflammatory response can cause neurotoxicity, which is the main source of inflammatory factors and oxidative stress [10–12]. Therefore, inhibition of microglial inflammation is of great significance for neuroinflammation. Lipopolysaccharide (LPS) is a component of bacterial cell walls. Studies have shown that LPS can activate multiple TLR4 receptors and cause the inflammatory response of the cells [13, 14]. Therefore, LPS was used to stimulate BV2 cells to establish a microglia inflammation cell model.

*β*-Sitosterol (BS) is one of the phytosterols and belongs to the tetracyclic triads. It is widely found in plant seeds such as various vegetable oils and nuts in nature, as well as in certain plant drugs [15–17]. At present, *β*-sitosterol is widely used in the pharmaceutical industry due to its unique biological properties and physicochemical properties. Studies have shown that *β*-sitosterol has a wide range of anti-inflammatory effects in peripheral tissues. It has a certain role in inflammation models such as chronic obesity-related inflammation, ovalbumin-induced lung inflammation, TNBS-induced colitis, and rheumatoid inflammation in mice [18–20]. However, it has not been reported whether it also exerts an anti-inflammatory effect on microglia inflammation. Therefore, this experiment aims at studying the effect of *β*-sitosterol on microglia activity and its mechanism.

## 2. Materials and Methods

### 2.1. Materials and Reagents

Beta-Sitosterol were obtained from Chengdu Pufei De Biotech (>98% purity; Pufei de, Chengdu, China). Trizol, LPS (from Escherichia coli), and Dimethyl sulfoxide (DMSO) were obtained from Sigma-Aldrich (MO, USA). The 0.05% trypsin was obtained from MBX Biotechnologies (Fuzhou, China). Dulbecco's modified Eagle's medium (DMEM) and fetal bovine serum (FBS)) were purchased from Invitrogen (Carlsbad, CA, USA). The PrimeScript® 1st Strand cDNA Synthesis Kit was purchased from Roche (South San Francisco, CA, USA). The SYBR Green QuantiTect RT-PCR Kit was purchased from Takara Biotechnology (Ltd., Kyoto, Japan). The TNF-*α*, IL-6, and IL-1*β* ELISA kits were purchased from R&D Systems (Abingdon, UK).

### 2.2. Cell Culture

BV2 cells, mouse microglia cell line, were purchased from the Cell Culture Center at the Institute of Basic Medical Sciences, Chinese Academy of Medical Sciences (Beijing, China). The cells were cultivated in DMEM including 10% FBS, 50 *μ*g/mL streptomycin, and 50 U/mL penicillin (incubator conditions: 37°C and 5% CO_2_). The BV2 was replaced fresh medium with serum every day and passaged at approximately 80% confluence.

### 2.3. CCK-8 Assay

The effect of BS on cell viability was detected by CCK-8 assay. The experiment was divided into 6 groups, without treatment group (control), DMSO treatment group, and different concentration BS (2, 4, 8, and 16 *μ*M) treatment group. Briefly, growing-well BV2 cells (1 − 1.5 × 10^3^/well) were inoculated in a 96-well plate. After the cells were cultured for 24 h, the different concentrations of BS (2, 4, 8, and 16 *μ*M) were added to the wells and incubated for 20 h. After adding to CCK-8 (Sigma (St. Louis, MO, USA)) for 2 h, the absorbance was measured with a microplate reader at 450 nm.

### 2.4. Real-Time PCR Assay

The well-growing BV2 were inoculated in a 6-well plate and replaced with serum-free DMEM for 3 h when the cell concentration was up to 80%, then treated with different concentrations of BS for 1 h before LPS (100 ng/mL) induction. After LPS induction for 6 h, total RNA was extracted with trizol reagent and reversed it to cDNA using a RT-PCR Kit. Then, the levels of the gene expression were measured by SYBR Green QuantiTect RT-PCR Kit and evaluated three times per sample. The cDNA levels of each sample were computed relative to *β*-action by the comparative cycle threshold method. The primer sequences of gene expression were presented in Table 1.

### 2.5. Enzyme-Linked Immunosorbent Assay (ELISA)

The well-growing BV2 cells were inoculated in a 6-well plate and cultured at the CO_2_ incubator. When concentration reached about 50%, the cells were pretreated with BS for 2 h and exposed to LPS (100 ng/mL) for another 24 h. Then, the supernatant was gathered and the protein levels of IL-6; TNF-*α* were tested through the ELISA kits.

### 2.6. Western Blot Analysis

Well-growing BV2 is inoculated in a cell culture dish 6 cm in diameter. When concentration was up to 80%, the cells were processed with BS and LPS for a certain time. After that, the cell precipitation was collected and cracked in RIPA lysis buffer. The protein concentration was measured using a bicinchoninic acid protein assay kit. A total of 40 *μ*g of protein was subjected to 13% sodium dodecyl sulfate-polyacrylamide gel electrophoresis (SDS-PAGE) and transferred onto polyvinylidene difluoride membranes (PVDF) (Amersham Pharmacia Biotech, Tokyo, Japan). After blocking with 5% nonfat milk for 3 h at room temperature, the membranes were incubated over 12 h at 4°C with primary antibodies against *β*-actin (1 : 10,000) (Santa Cruz, CA, USA), iNOS (1 : 4000), COX-2 (1 : 4000), p-ERK (1 : 2000), ERK (1 : 5000), p-p38 (1 : 2000), p38 (1 : 5000), p-JNK (1 : 2000), JNK (1 : 4000), p-NF-*κ*B p65 (1 : 5000), and NF-*κ*B p65 (1 : 2000) (Cell Signaling Technology, Danvers, MA, USA) and incubated at room temperature for 1 h against the secondary antibodies goat anti-rabbit (1 : 5000) or goat anti-mouse (1 : 5000). The proteins were tested using ECL Western blotting Detection Reagents (South San Francisco, CA, USA).

### 2.7. Primary Microglia Culture

Primary microglia cells were obtained from newborn to 24 h old SD rats. In short, the brains of newborn rats were placed into PBS. The meninges were removed, and the cerebral cortex and midbrain tissue are separated. Then tissues were shredded (less than 1 mm^3^) and digested in 0.25% trypsin for 15 min at 37°C. Then, we added DMEM supplemented with 10% FBS to stop digestion. After that, the cell suspension was filtered through a 40 *μ*m mesh. Then, the cells were cultured in cell culture flask with DMEM supplemented with 10% FBS. Half of the culture media was changed every 2 days. After 14 days, primary microglia were harvested by shaking the flask for 4 h at 100 rpm and then seeded onto new plates precoated with PLL.

### 2.8. Statistical Analysis

The data were presented in the form of mean ± SD. The results were analyzed with SPSS 14.0. The differences between the groups were evaluated with ANOVA. The *p* < 0.05 was considered to be statistically significant.

## 3. Results

### 3.1. Effect of BS on BV2 Cell Growth

**Figure 1:**
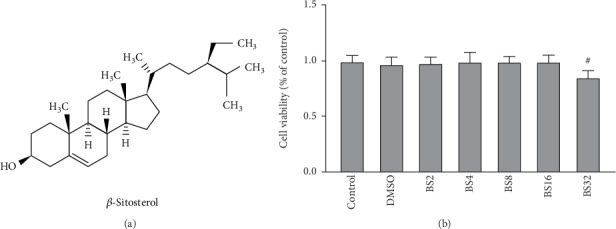
(a) The chemical structure of *β*-sitosterol (BS). (b) The effect of BS on the viability of BV2. BV2 cells are seeded in 96-well plates at a density of 1 − 3 × 10^4^/mL. Then, the cells were treated for 18 h with different concentrates of BS (0, 2, 4, 8, and 16 *μ*M) prior to the detection of the cell viability. The results were presented as mean ± SD (*n* = 10). ^#^*p* < 0.05 versus the control group.

To measure whether BS affects the growth of BV2, CCK-8 experiment was performed to test the viability of BV2. After BV2 was exposed to different concentrations of BS (0, 2, 4, 8, and 16 *μ*M) for 24 hours, the CCK-8 solution was added to the culture wells. After 2 h, the results were measured at 450 nm. As shown in Figure 1, BS(0-16 *μ*M) had no significant toxicity to BV2 cells. BS (32 *μ*M) treatment resulted in a reduced survival rate of BV2 cells (Figure 1(b)). Therefore, we chose two concentrations of 8 and 16 *μ*M for subsequent studies.

### 3.2. BS Inhibits the mRNA Levels of LPS-Induced Proinflammatory Mediators in BV2 Cells

**Figure 2:**
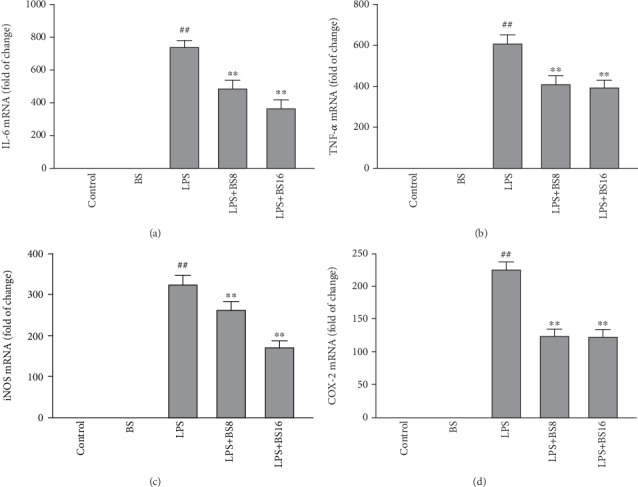
The effect of BS on the mRNA expression of proinflammatory mediators in BV2 cells. The cells were pretreated with BS for 2 h prior to the exposure of LPS (100 ng/mL); after 12 h, the cells were collected and the mRNA levels of proinflammatory mediators (IL-6 (a), TNF-*α* (b), iNOS (c), and COX-2 (d)) were tested by real-time PCR. The results were presented as mean ± SD (*n* = 4). ^##^*p* < 0.01 versus the control group. ^∗∗^*p* < 0.01 and ^∗^*p* < 0.05 versus the LPS-stimulated group.

To study whether BS can inhibit microglia inflammation, we studied the effect of BS on the mRNA levels of proinflammatory mediators (IL-6, TNF-*α*, iNOS, and COX-2). Firstly, well-growing BV2 were preprotected with BS for 1 h and exposed to LPS (100 ng/mL) for 6 h then the cells were collected to detect mRNA levels of proinflammatory mediators. The results in Figure 2 showed that LPS exposure can significantly increase the mRNA expression levels of proinflammatory mediators (IL-6 (a), TNF-*α* (b), iNOS (c), and COX-2 (d)), while BS treatment can significantly inhibit this effect.

### 3.3. BS Inhibits the Protein Levels of LPS-Induced Proinflammatory Mediators

**Figure 3:**
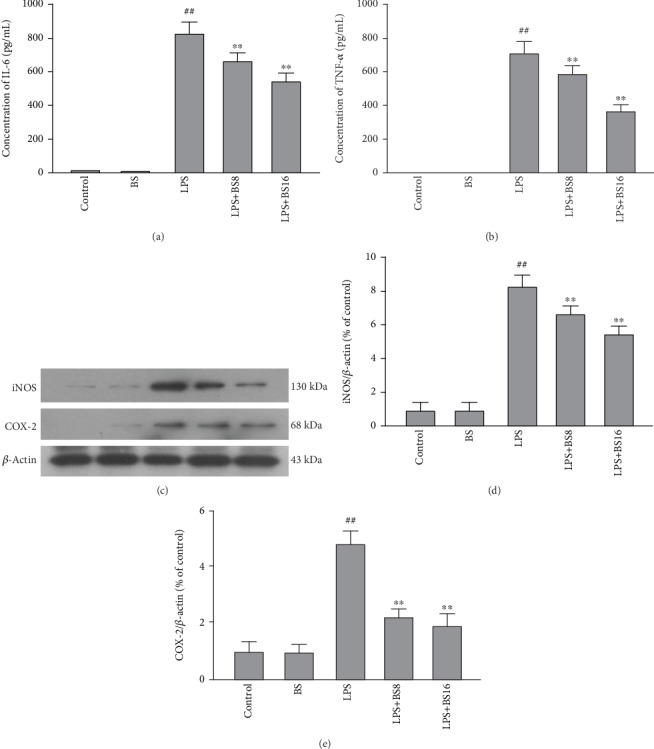
The effect of BS on the protein expression of proinflammatory mediators in BV2 cells. The cells were pretreated with BS for 2 h prior to the stimulation of LPS (100 ng/mL). After 24 h, the cells and the supernatant were collected, then the protein levels of proinflammatory mediators were tested by ELISA (IL-6 (a) and TNF-*α* (b)) and western blot (iNOS and COX-2)(c–e). The results were presented as mean ± SD (*n* = 4). ^##^*p* < 0.01 versus the control group. ^∗∗^*p* < 0.01 and ^∗^*p* < 0.05 versus the LPS-stimulated group.

The mRNA is known to guide protein translation, and proteins perform a variety of functions. In order to further clarify the part of BS in inhibiting inflammation, we also studied the influence of BS on the protein levels of proinflammatory mediators (IL-6, iNOS, COX-2, and TNF-*α*). Firstly, well-growing BV2 were pretreated with BS for 1 h and exposed to LPS (100 ng/mL) for 12 h; then, the supernatant was collected to detect the protein levels of proinflammatory factors (IL-6 and TNF-*α*) by ELISA and cells pellet was collected to detect the protein levels of the proinflammatory enzyme (iNOS and COX-2) by western blotting. The results in Figure 3 showed that BS can inhibit the LPS-induced protein levels of proinflammatory mediators (IL-6 (a), TNF-*α* (b), iNOS (c, d), and COX-2 (c, e)) in BV2 cells.

### 3.4. BS Represses the LPS-Induced Activation of p38, ERK1/2, and NF-*κ*B Pathway in BV2 Cells

**Figure 4:**
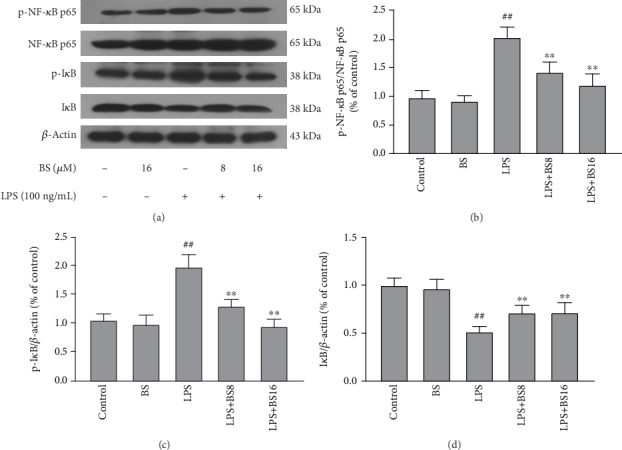
The effect of BS on the activation of the NF-*κ*B pathway. The cells were pretreated with BS for 2 h prior to the stimulation of LPS (100 ng/mL). After 2 h, the cell pellet was collected and extracted the total protein. After that, the expression levels of NF-*κ*B p65 (a, b), p-NF-*κ*B p65 (a, b), I*κ*B (a, d), p-I*κ*B (a, c), and *β*-actin were detected by western blot. The results were presented as mean ± SD (*n* = 4). ^##^*p* < 0.01 versus the control group. ^∗∗^*p* < 0.01 and ^∗^*p* < 0.05 versus the LPS-stimulated group.

**Figure 5:**
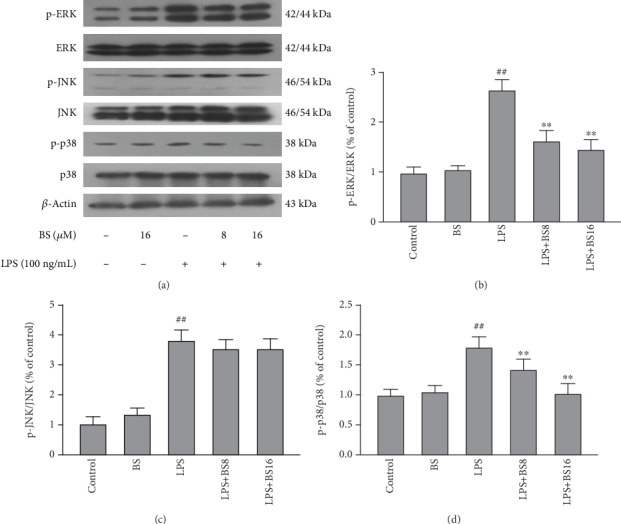
The effect of BS on the activation of the MAPK pathway. The cells were pretreated with BS for 2 h prior to the stimulation of LPS (100 ng/mL). After 2 h, the cell pellet was collected and extracted the total protein. After that, the expression levels of p-ERK, ERK (a, b), p-JNK, JNK (a, c), p-p38, p38(a, d), and *β*-actin were detected by western blot. The results were presented as mean ± SD (*n* = 4). ^##^*p* < 0.01 versus the control group. ^∗∗^*p* < 0.01 and ^∗^*p* < 0.05 versus the LPS-stimulated group.

NF-*κ*B and MAPKs are two classic inflammatory pathways. Numerous studies have shown that the NF-*κ*B and MAPK pathways play a crucial role in the inflammation process, and they can regulate the transcription of multiple inflammatory mediators when activated. To clarify the anti-inflammatory mechanism of BS, we studied the influence of BS on NF-*κ*B (Figure 4) and MAPK (Figure 5) pathway. Firstly, well-growing BV2 were pretreated with BS for 1 h and exposed to LPS (100 ng/mL) for 2 h then cells pellet was collected to detect the protein expression levels of NF-*κ*B and MAPK pathway key molecules by western blot.

### 3.5. BS Inhibits the mRNA Levels of INF*γ*-Induced Proinflammatory Mediators in BV2 Cells

**Figure 6:**
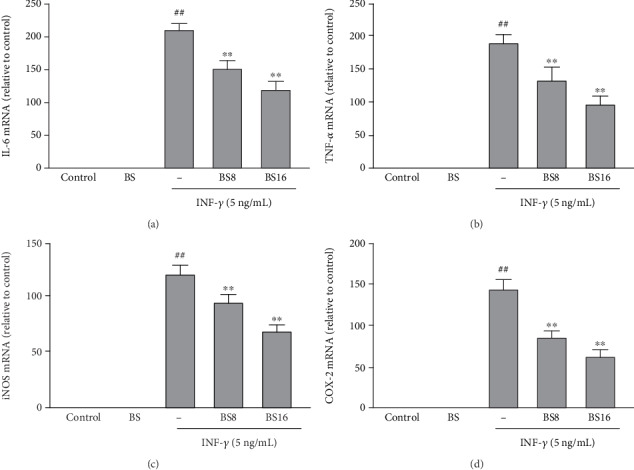
The effect of BS on the mRNA expression of proinflammatory mediators in INF*γ*-exposed BV2 cells. The cells were pretreated with BS for 2 h prior to the exposure of INF*γ* (5 ng/mL); after 12 h, the cells were collected and the mRNA levels of proinflammatory mediators (IL-6 (a), TNF-*α* (b), iNOS (c), and COX-2 (d)) were tested by real-time PCR. The results were presented as mean ± SD (*n* = 4). ^##^*p* < 0.01 versus the control group. ^∗∗^*p* < 0.01 and ^∗^*p* < 0.05 versus the INF*γ*-stimulated group.

To verify the inhibitory effect of BS on aseptic inflammation, we investigated the effect of BS on mRNA expression of INF*γ*-induced proinflammatory mediator (IL-6, TNF-*α*, iNOS, and COX-2) in BV2 cells using real-time PCR. The results in Figure 6 showed that BS can significantly inhibit the release of proinflammatory mediators (IL-6 (a), TNF-*α* (B), iNOS (c), and COX-2 (d)) in INF*γ*- exposed BV2 cells.

### 3.6. BS Inhibits the mRNA Levels of LPS-Induced Proinflammatory Mediators in Primary Microglia Cells

**Figure 7:**
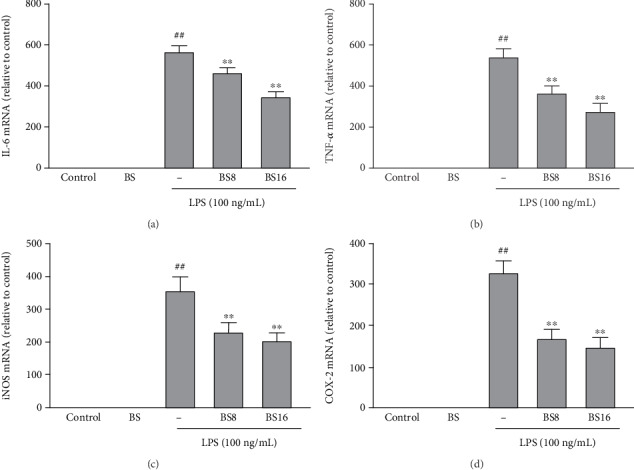
The effect of BS on the mRNA expression of proinflammatory mediators in primary microglia. The cells were pretreated with BS for 2 h prior to the exposure of LPS (100 ng/mL); after 12 h, the cells were collected and the mRNA levels of proinflammatory mediators (IL-6 (a), TNF-*α* (b), iNOS (c), and COX-2 (d)) were tested by real-time PCR. The results were presented as mean ± SD (*n* = 4). ^##^*p* < 0.01 versus the control group. ^∗∗^*p* < 0.01 and ^∗^*p* < 0.05 versus the LPS-stimulated group.

To further verify the effect of BS on microglial inflammation, we purchased primary microglia and cultured them in 6-well plates. Then, the effects of BS on LPS-induced microglia inflammatory response were investigated using real-time PCR. The results in Figure 7 showed that BS can significantly inhibit the mRNA expression of proinflammatory mediators (IL-6 (a), TNF-*α* (b), iNOS (c), and COX-2 (d)) in LPS-exposed microglia.

## 4. Discussion

In this study, we found that BS treatment inhibited the production of proinflammatory mediators (IL-6, iNOS, COX-2, and TNF-*α*) in microglia, and further mechanism studies found that BS treatment repressed LPS-induced phosphorylation and degradation of I*κ*B and repressed the phosphorylation of signal molecules such as NF-*κ*B p65, p38, and ERK. It was concluded that BS can inhibit the inflammation of BV2 cells exposed to LPS by inhibiting the activation of ERK, p38, and NF-*κ*B pathway.

Current researches have confirmed that inflammation plays a vital part in brain diseases. When the body is stimulated by some harmful factors, autoimmunity is activated, and immune cells respond quickly, and the body monitors, clears, and protects the body through a slight inflammatory response [21–23]. However, the persistence of the inflammatory response will cause a large amount of toxic factors such as PGE2, NO, proinflammatory mediators to accumulate in the central nervous system, which will seriously damage peripheral neurons and cause neurodegenerative diseases [7, 24, 25]. Therefore, inhibiting the release of toxic mediators can protect neurons and relieve neurodegenerative diseases. Microglia is immune cells in the brain. Under normal physiological conditions, microglia is at rest. Once activated, they can release a large amount of chemokines and proinflammatory mediators, causing continuous damage to the body [26–28]. Therefore, many studies have focused on inhibiting microglial inflammation in order to provide new ideas for the treatment of neurodegeneration diseases.

In the current study, BS inhibits the production of proinflammatory mediators in BV2, however its anti-inflammatory mechanism is unclear. Many articles have reported that NF-*κ*B and MAPK play a vital part in the inflammatory response and immune response of cells [29, 30]. NF-*κ*B is caused by extracellular stimulation. Extracellular signal factors bind to receptors on the cell membrane, initiating a series of downstream reactions [31^,^^32^]. The receptor protein activates I*κ*B kinase (IKK) after being stimulated. IKK phosphorylates serine at the regulatory site of the I*κ*B subunit of the NF-*κ*B I*κ*B complex in cells, causing the I*κ*B subunit to be modified by ubiquitination and then degraded by proteases to release the NF-*κ*B dimer. Free NF-*κ*B enters the nucleus and combines with genes with NF-*κ*B binding sites to initiate the transcription process [33–35]. In the study, we found that BS treatment can significantly inhibit LPS-mediated degradation and phosphorylation of I*κ*B and inhibit phosphorylation of NF-*κ*B p65 in microglia. The MAPK pathway is one of the common intersection pathways of signal transduction pathways such as cell proliferation, stress, inflammation, and apoptosis. In different cells, different signal pathways constrained by different cytoskeleton can produce multiple effects [36, 37]. The MAPK pathway mainly includes three subunits: ERK, JNK, and P38. ERK exists widely in various tissues and is involved in the regulation of cell proliferation and differentiation. The JNK family is a key molecule for various stressors to induce cell signal transduction and participates in cells' stress response to radiation, osmotic pressure, and temperature changes. P38 mediates inflammation, apoptosis, etc., thus becomes a target for the filter of anti-inflammatory drugs [38–40]. In the current study, we found that BS treatment can significantly inhibit LPS-mediated activation of P38 and ERK without significantly affecting the JNK pathway activation in microglia.

In summary, our experiments have initially verified that BS has anti-inflammatory effects on microglia and found that BS exerts anti-inflammatory effects mainly by inhibiting the activation of P38, ERK, and NF-*κ*B pathways. As one of the plant components, BS has the potential to be developed as an anti-inflammatory drug due to its wide source and nontoxic natural properties. It is also of great significance to further study the role of BS. Further research may focus on the effects of BS in vivo and its more specific anti-inflammatory mechanisms.

## Figures and Tables

**Table 1:** The primer sequences of *β*-actin, iNOS, COX-2, TNF-*α*, IL-1*β*, and IL-6.

Gene	Sequences	Length (bp)
*β-Action*	(F)50-GTCAGGTCATCACTATCGGCAAT-30 (R)50-AGAGGTCTTTACGGATGTCAACGT-30	147
*COX-2*	(F) 50-AGAGTCAGTTAGTGGGTAGT-30 (R) 50-CTTGTAGTAGGCTTAAACATAG-30	170
*iNOS*	(F) 50-CACCCAGAAGAGTTACAGC-30 (R) 50-GGAGGGAAGGGAGAATAG-30	186
*IL-6*	(F) 50-AGCCACTGCCTTCCCTAC-30 (R) 50-TTGCCATTGCACAACTCTT-30	156
*TNF-α*	(F) 50-CCACGCTCTTCTGTCTACTG-30 (R) 50-GCTACGGGCTTGTCACTC-30	145

## Data Availability

All the data used to support the findings of the study are available from the corresponding author upon request.
